# Anchors as Semantic Primes in Value Construction: An EEG Study of the Anchoring Effect

**DOI:** 10.1371/journal.pone.0139954

**Published:** 2015-10-06

**Authors:** Qingguo Ma, Diandian Li, Qiang Shen, Wenwei Qiu

**Affiliations:** 1 School of Management, Zhejiang University, Hangzhou, China; 2 Neuromanagement Lab, Zhejiang University, Hangzhou, China; National University of Singapore, SINGAPORE

## Abstract

Previous research regarding anchoring effects has demonstrated that human judgments are often assimilated to irrelevant information. Studies have demonstrated that anchors influence the economic valuation of various products and experiences; however, the cognitive explanations of this effect remain controversial, and its neural mechanisms have rarely been explored. In the current study, we conducted an electroencephalography (EEG) experiment to investigate the anchoring effect on willingness to accept (WTA) for an aversive hedonic experience and the role of anchors in this judgment heuristic. The behavioral results demonstrated that random numbers affect participants’ WTA for listening to pieces of noise. The participants asked for higher pay after comparing their WTA with higher numbers. The EEG results indicated that anchors also influenced the neural underpinnings of the valuation process. Specifically, when a higher anchor number was drawn, larger P2 and late positive potential amplitudes were elicited, reflecting the anticipation of more intensive pain from the subsequent noise. Moreover, higher anchors induced a stronger theta band power increase compared with lower anchors when subjects listened to the noises, indicating that the participants felt more unpleasant during the actual experience of the noise. The levels of unpleasantness during both anticipation and experience were consistent with the semantic information implied by the anchors. Therefore, these data suggest that a semantic priming process underlies the anchoring effect in WTA. This study provides proof for the robustness of the anchoring effect and neural evidence of the semantic priming model. Our findings indicate that activated contextual information, even seemingly irrelevant, can be embedded in the construction of economic value in the brain.

## Introduction

The anchoring effect is a typical human decision heuristic as demonstrated by Tversky and Kahneman [1] in their seminal research. The anchoring effect describes a phenomenon in which judgment is biased toward an initially presented value, namely, the anchor. In contrast to the assumptions of traditional economic theories, it is implied that valuation can be influenced by arbitrary values. Researchers often use willingness to pay (WTP) and willingness to accept (WTA) as indices of valuation. WTP refers to the maximum amount of money one would be willing to offer for a good or experience and WTA represents the minimum compensation demanded for a person to forgo a good or bear some suffering/harm. Studies have demonstrated anchoring effects in consumer willingness to pay (WTP) and willingness to accept (WTA). In experimental settings, anchors affect WTP and/or WTA for consumer goods [2–7], public goods [8] and hedonic experience [2]. Field studies and real-world data analyses have also provided evidence of the omnipresence of anchoring effects [9,10]. In contrast to the substantial research demonstrating anchoring effects in WTP and/or WTA for common products, the limited studies of anchoring effects in WTA for hedonic experiences have presented inconsistent results and casted doubts on the robustness of this phenomenon [2,11–14]. Therefore, further evidence on the robustness of anchoring effects in hedonic experience valuation is needed.

Studies in the field of WTA/WTP mainly focus on the presence and robustness of the anchoring effect and its moderators [7,8,15,16], and discussion of the influence of anchors on valuation judgments has been limited. Previous research suggests that various anchoring effects are mainly produced by two mechanisms: insufficient adjustment from the initial value [1,17,18] and semantic priming by anchors [19–21]. It is also suggested that the two sources of anchor value, i.e., self-generated and externally provided, are responsible for the adjustment and the semantic priming, respectively [17,18]. Some studies of WTP and/or WTA for products have implied that these two mechanisms exert an independent or interactive influence on anchoring effects [3,5,22,23]. However, the effect of anchors on WTP and WTA for hedonic experiences still requires further discussion and evidence. Furthermore, while there have been substantial discussions of the psychological mechanisms of anchoring effects, the neural underpinnings of anchored processes have received less attention [24,25]. Among the few neuroscientific studies regarding anchoring effects, Qu et al. [24] employed a “dot-image” paradigm and provided event-related potential (ERP) evidence for the insufficient adjustment model in the situation of simple physical property judgment. Tamir and Mitchell [25] posited that people use their own thoughts and feelings as starting points when making inferences about others’ preferences or traits. In their functional magnetic resonance imaging (fMRI) study, the activation of medial prefrontal cortex was linear correlated with self-other discrepancy of the mental state judgment. The results suggested an “anchoring-and-adjustment” process underlying such a social cognition phenomenon. These few studies have helped to disentangle the conflicts of explanations of anchoring effects in several judgment domains. The neurocognitive processes involved in anchoring effects on valuation, nevertheless, have not been examined.

In this research, we investigated how arbitrary information, i.e., an anchor, influences the construction of value in aversive hedonic experiences and the electrophysiological correlates of this impact on the cognitive process. The goals of the present study were two-fold. First, we investigated the robustness of anchoring effects in the valuation of hedonic experiences, particularly in the WTA for an aversive experience, since more doubt has been cast on the prevalence of anchoring effects in WTA compared to WTP [3,4]. We conducted a WTA task very similar to that described by Ariely et al. [2], in which subjects listened to sample pieces of noise, compared their WTA for listening to the same noise at a greater intensity with a random number, and ultimately provided their WTA.

Second, given that arbitrary anchors have exhibited an effect on subjects’ WTA for hedonic experiences, we attempted to identify the mental process involved in this type of anchored valuation by providing corresponding neural evidence. The anchors were drawn from an outside source in our paradigm. Based on studies on anchor sources [17,18], these anchors likely activate accessible semantic information used for valuation. This semantic priming process is consistent with the “expectation and ‘perceptual’” model [26] for human value construction. This model assumes that individuals make inferences from contextual information (including anchors), which then influences their experience with the judging targets. Related neuroscience studies of the context-dependency of valuation have validated this model and indicate that external references or labels with valuation targets can alter expectations and/or experiences in value construction processes, which are reflected in brain activities [27–34]. Therefore, we hypothesized that an anchor in our study should act as a hint/cue recognized by the subjects that would influence both their belief prior to listening and their sensory experience while listening to the noise, thus eventually biasing their WTA. In other words, the subjects would be primed by the arbitrary anchors, and their expectations and real hedonic experiences would be assimilated to what the anchors semantically indicated. A difference in the anchor-consistent semantic information activated by higher or lower anchors would produce a discrepancy in the subjective feelings of the aversiveness of the noise. Furthermore, the neural activity would reflect this priming process by manifesting emotions during expectancy and real experience consistent with the anchors’ semantic labels. A larger anchor number perceived as a cue that preceded a more painful audio stimulus should elicit electroencephalography (EEG) responses indicating the anticipation of a more unpleasant experience. In addition, the EEG underpinnings of valuation formation when the noise was played should indicate that the subjects feel the noise as less tolerable after a larger number. Previous findings suggested that anticipation to various aversive or unpleasant events is associated with ERP components such as P2 and late positive complex (usually including P3 and late positive potential (LPP)). Cues of electrical shock threats or upcoming negative affective pictures usually induce larger P2 amplitude over frontal lobe and enhanced P3 or LPP over more posterior regions [35–39]. Since annoying sounds in our experiment were comparable to shocks or negative pictures in the way that they were all hedonic experience which are sensorially or emotionally aversive, we could expect to see difference of these ERP components if different anchor numbers were perceived as different cues to the following sounds. Besides the studies on phase locked ERP components, researchers have also delved into the non-phase locked neural oscillations involved in various cognitive processes (see reviews: [40,41]. Event-related oscillatory activities are characterized as relative power increase or decrease in certain frequency bands caused by event-related synchronization (ERS) or desynchronization (ERD) [42]. Studies taking the perspective of event-related band power have shown that theta band ERS is associated with processing of emotional stimuli. Larger theta band ERS is proved to be associated with higher arousal [43–46] and relatively negative valence in emotion [47] or negative appraisal [48]. If the anchors in our study did modulate subjects’ perception on the painfulness of the noise, then theta band power, which is a highly probable EEG correlate of emotional valence and arousal, would also differ in whether subjects were primed by high or low anchors.

## Materials and Methods

### Ethics Statement

This study was approved by the Internal Review Board, Neuromanagement Lab, Zhejiang University. All participants were fully informed of the study protocol and provided written consent.

### Participants

Twenty-four subjects were recruited for the experiment (10 females, 14 males; mean age: 22.68 years old, standard deviation (SD) = 2.60). They were registered students of Zhejiang University. All participants were right handed by self-report, had normal or corrected to normal vision and reported no history of neurological or mental diseases. One subject reported feeling itchy on her scalp during the EEG recording and withdrew from the study. Two subjects quitted the study because the experiment procedure could not be implemented based on their behavior in the pilot session (see: the Stimuli and procedure section). Other two subjects were also excluded from data analysis because of substantial EEG artifacts. As a result, the following analysis was performed using the data obtained from 19 (7 females) subjects.

### Stimuli and procedure

We adopted a design that was similar to but modified from the tasks in the research of Ariely et al. [2]. In the current study, the subjects would first sample a piece of noise in each trial and then provide their WTA for undergoing the noise in an intensified volume. They would see a random anchor number from the high or the low anchor experiment condition in each trial and compare this number with the WTA before providing the final answer.

#### Noise stimuli

Each of the noise pieces used in the experiment lasted 4 seconds and was constructed by a two-step process. First, two 4-second-long dissonant sound waves were generated using oscillator functions in MATLAB R2008b (MathWorks, Massachusetts, USA). Second, the waves were synthesized by an audio recording/processing software WaveCN (http://wavecn.com/, China). By this process we had a dataset with 80 candidate noise pieces and those 45 pieces of noise played in the main session of our experiment were selected from this dataset. The selection was based on the ratings of the annoyance of each candidate noise piece on a 9-point Likert scale (1 = not annoying, 9 = extremely annoying) by a calibration group of 48 participants. The annoyance of each noise piece in the candidate set was computed by averaging the ratings of all participants (mean = 5.08, SD = 0.57). The final set comprised the sound pieces with the least variances, i.e., the smallest SD, on the annoyance rating. This procedure ensured that the sound pieces were annoying (mean = 5.24) and similar in annoyance (SD = 0.48) to avoid confounding effects of differences in annoyance.

#### Pilot and anchor value generation

In our experiment, a within-subject design was used for the concerned factor (anchor: high vs. low). Anchor values, though arbitrary, were selected using certain rules. As stated by Tufano [11], noise is a “private-value good”. Each subject’s tolerance and sensitivity to noise is different, and the same sound piece might induce different levels of unpleasantness. Therefore, the average WTA should also vary among the subjects. Thus, it was important that the anchor values in the high and low conditions were generated based on an individual subject’s WTA range prior to the main session. In this pilot session, the subjects were asked to provide their WTA for 10 pieces of noise from the sound dataset without viewing any anchors, and this session was instructed as a practice of the pricing task because it was quite novel to the subjects. The mean (*mean*_*pilot*_) and SD (*SD*_*pilot*_) of WTA of each subject were obtained from this pilot session to compute the individual intervals from which high, medium and low anchors in the main session were drawn. The high anchors were 40 random integers from an interval of

$$Range_{high}=\{\begin{array}{c} \left[mean_{pilot}+2SD_{pilot},\;mean_{pilot}+4SD_{pilot}\right]\\ \left[mean_{pilot}\;2SD_{pilot},\;30\right],\;if\;mean_{pilot}+4SD_{pilot}>30 \end{array}$$ (1)

and the low anchors were also 40 random integers drawn from an interval of

$$Range_{low}=\{\begin{array}{c} \left[mean_{pilot}-4SD_{pilot},\;mean_{pilot}-2SD_{pilot}\right]\\ \left[1,\;mean_{pilot}-2SD_{pilot}\right],\;if\;mean_{pilot}-4SD_{pilot}<1 \end{array}$$ (2)

To ensure the randomly drawn integers were more successive and thus natural to the subjects, 10 medium random anchors were also drawn from an interval of

$$Range_{medium}=\left[mean_{pilot}-SD_{pilot},\;mean_{pilot}+SD_{pilot}\right]$$ (3)

However, the trials with the medium anchors were not used for the subsequent EEG analysis. An upper limit (Chinese Yuan (CNY) ¥30) of the price range was set because of the limitation of the experimental procedure, which will be explained later. The subjects were instructed that we set an upper limit of CNY ¥30 because we did not have additional money to compensate their unpleasant experiences; however, it was fair if they asked for more than CNY ¥30. In this case, they could provide an answer equal to 30. Given the rules of anchor generation, two subjects did not complete the experiment and were excluded from the study. The upper limits of their *Range*_*low*_ were negative; thus, the experimental design could not be implemented.

#### Experimental task in the main session

The main session consisted of 90 trials (high: 40; medium: 10; and low: 40). In each trial, the subjects listened to a piece of noise and provided their WTA for listening to this same noise at quadruple volume. They also fully understood that after all 90 trials, only one trial would be randomly drawn and counted and that every single trial should be treated seriously because any trial might be selected. For the later chosen noise piece, a Becker-DeGroot-Marschak auction was used to determine if they had to endure the unpleasantness and obtain the compensation or experience a no pain, no “compensate” situation [49]. Following this rule, the WTA of the chosen trial was compared with a random integer N written on a price tag drawn by the subject from a carton. N followed a triangle-distribution that ranged from CNY ¥1 to CNY ¥15 [2]. If WTA ≤ N, the subject would listen to the same noise with an intensified volume and receive a compensation equal to N. By contrast, if WTA > N, the subject would leave. This auction was adopted to ensure and motivate the subjects to provide the true WTA [50]. The optimal strategy of providing the true WTA was explained in detail to the subjects.

**Fig 1:**
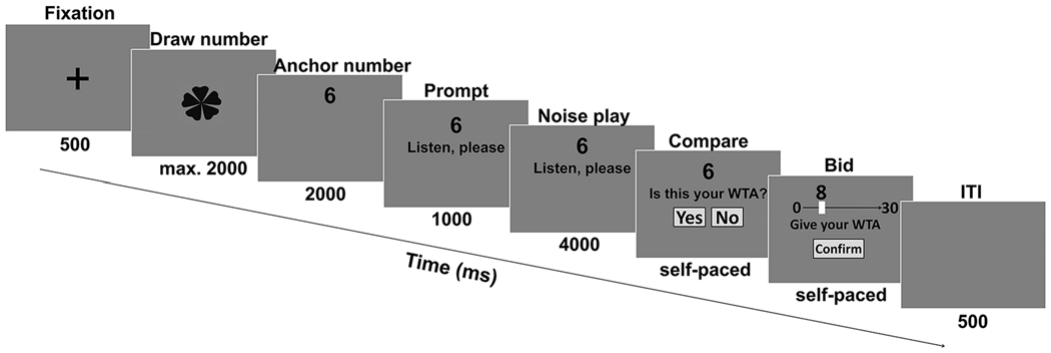
Experimental paradigm in the main session. Subjects first drew a random number and then listened to a piece of noise. After comparing their WTA with the drawn number, they reported their WTA.

During the main session, the participants sat in a comfortable chair in front of and approximately 1 meter away from a 17-inch CRT screen and in an electrically shielded and acoustically isolated room, while their EEG was simultaneously recorded. The stimuli were presented by the E-Prime 2.0 Software Package (Psychology Software Tools, Pittsburgh, USA) with a maximum visual angle of ~18.4° × 13.6°. The paradigm is illustrated in Fig 1. During each trial, a fixation cross was presented for 500 ms. Next, similar to the “spinning wheel” used in Tversky and Kahneman [51], a rotating clover was shown on the screen, and the subjects could click the mouse at any time within 2 seconds to draw a random number from the computer. This rotating clover was explained as a “spinning wheel” mechanism to the subjects in the experiment instruction. After a random interval between 800 and 1200 ms after the mouse click, the anchor number was shown for 2000 ms, followed by a 1000-ms prompt phrase for the upcoming noise during which the number continued to be displayed. Then, after listening to the noise for 4000 ms, the subjects were asked to judge the unpleasantness that resulted from that noise and decide the lowest amount of monetary compensation they would accept for listening to the same noise at quadruple volume. When the noise ended, the subjects compared and answered whether the previously drawn number was equal to their WTA and clicked on the “Yes” or “No” button. Finally, they clicked on a price axis with a range of 1 to 30, moved the cursor to the position that represented their WTA, and clicked the “Confirm” button to submit (see: experimental instruction in S1 Text, for the instruction of the whole procedure to the subjects). The inter-trial interval was 500 ms. We investigated multiple ways to obtain the subjects’ WTA input via several pilot studies and adopted this “cursor-on-axis” method. This method was chosen because pressing the numeric keyboard in their hand proved to be difficult for the subjects because they had to continue to watch the screen and because pressing the keyboard to increase or decrease the price from the anchor may create the confusion of a status quo bias [52] in the experiment. For a viable procedure, the numeric axis could not be unlimited; thus, we set an upper limit of 30 CNY based on a WTA range from a preliminary experiment. The noise piece played in each trial was randomly chosen and assigned to a certain anchor condition. Forty-five noise pieces other than the pieces used in the pilot session were played twice in the main session.

### EEG recording

EEG was continuously recorded with a Neuroscan Synamp2 Amplifier (Scan 4.3.1, Neurosoft Labs, Inc. Virginia, USA) with 64 Ag/AgCl electrodes positioned according to the International 10–20 System (band pass: 0.05–100 Hz; sampling rate: 1000 Hz). All electrodes were first referenced to the left mastoid and later digitally re-referenced to the linked mastoids. Vertical and horizontal electrooculograms (EOGs) were recorded with two pairs of electrodes. One pair was placed above and beneath the left eye in parallel with the pupil, and the other was placed at the outer canthus of each eye. All electrode impedance was maintained below 5 kΩ. The offline processing of the vertical ocular artifact correction used the regression approach described by Semlitsch et al. [53]. The recorded data with blocks contaminated by amplifier clippings and bursts of electromyographic activity were rejected.

### Data analysis

#### WTA data

The WTAs provided by each subject were averaged within each anchor condition (high, medium and low). The WTAs in these three anchor conditions were analyzed using repeated-measures analyses of variance (ANOVAs). The Greenhouse-Geisser correction was applied for the violation of the sphericity assumption in ANOVA (uncorrected degrees of freedom are reported with corrected *p*-values and epsilon values (*ε*)), and multiple comparisons were corrected with the Bonferroni method when appropriate.

To test for the effect size of anchors in our experiment and compare it with those in the previous studies, we analyzed this index in two ways. First, we computed the effect size as a percentage using the method described in Simonsohn et al. [14]. Second, we measured the effect size with Cohen’s d (Cohen’s dav, see: [54], which is a commonly used metric in psychological research.

#### ERP data

The EEG recordings for the ERP analysis were digitally low-pass filtered below 30 Hz (24 dB/Octave) and were segmented into epochs of 1000 ms, from 200 ms before to 800 ms after the anchor number stimuli and the noise onset, respectively, with the pre-stimuli period as the baseline. The epochs with baseline-to-peak deflections that exceeded ± 80 μV were excluded from averaging. The averaged ERPs were then created for each electrode of each subject under both high and low anchor conditions.

Similar to previous research [38,55,56], we observed components of P2 (160–260 ms) in the frontal and central regions and LPP (350–600 ms) over the parietal and occipital lobes when the anchor numbers were displayed. We also identified typical cortical auditory evoked potentials (CAEPs) [57,58] as the N1-P2 complex (120–210 ms, peak-to-peak) from the frontal to occipito-parietal regions at noise onset. For the P2 and LPP components, we used the mean amplitudes of the previously mentioned time windows. For the N1-P2 complex, we first defined two time windows of 100 to 150 ms and 180 to 240 ms for N1 and P2, respectively, by visual inspection of each subject’s data and identified the individual time points of extrema in these windows. Second, the peak amplitudes of N1 and P2 were then computed by the root mean square maximum with an interval of 50 ms centered at the extrema time points. The N1-P2 complex peak-to-peak amplitude represented the difference between these root mean squares [58,59].

Based on the relevant literature and visual inspection of topographic maps, we identified each component’s region of interest (ROI) that demonstrated the waveforms of the component. To examine the effects of caudality and laterality, as well as the anchor condition, we focused our statistical tests on certain electrodes that provided a good scalp distribution within the ROI of each ERP component. The statistical analysis included 9 electrodes of F3, Fz, F4, FC3, FCz, FC4, C3, Cz, and C4 in the frontal and central regions for P2; 12 electrodes of CP3, CPz, CP4, P3, Pz, P4, PO3, POz, PO4, O1, Oz and O2 for LPP; and 15 electrodes of F3, Fz, F4, FC3, FCz, FC4, C3, Cz, C4, CP3, CPz, CP4, P3, Pz and P4 for the N1-P2 complex. Repeated-measures ANOVAs were performed to assess the effects of three factors, anchor (high, low), caudality (anterior to posterior) and laterality (left, middle, right), on these three components. The Greenhouse-Geisser correction was applied for the violation of the sphericity assumption in ANOVA (uncorrected degrees of freedom are reported with corrected *p*-values and epsilon values (*ε*)), and multiple comparisons were corrected with the Bonferroni method when appropriate.

#### Band power data

To explore the potential non-phase locked neural oscillations involved in the valuation of the unpleasantness created by the noise, we examined the event-related band power changes. For the purpose of the time-frequency analysis regarding band power, a band-pass filter between 1 and 40 Hz (24 dB/Octave) was applied. The epoched data from 500 ms before and 4000 ms after the onset of the noise was baseline-corrected on the pre-stimulus interval, and the epochs with baseline-to-peak deflections that exceeded ± 80 μV were excluded. A short-term Fast Fourier Transform (FFT) with a fixed Hanning window of 250 ms was used for the spectrogram. We used ERS and ERD to characterize the power changes [42]. ERS/ERD was computed as the percentage of increase/decrease of power during the post-stimulus interval compared with baseline from -500 to -100 ms preceding noise onset [60]. The averaged ERS/ERD was obtained for each electrode of each subject under each condition (high and low).

A pronounced power increase between 1 and 9 Hz was identified shortly after noise onset (~80–280 ms) from the frontal to parietal regions. Similar to previous research regarding the emotion induced by and appraisal of various stimuli [45,47,48], a theta band ERS was observed when the subjects listened to the noises. Following an inspection of the electrodes’ time-frequency maps and the scalp topography of the band power change, we analyzed the theta band (4–8 Hz) ERS on 45 electrodes within the above time window. Similar to the methods for ERP statistics, the ERS data for these electrodes were analyzed according to their caudality and laterality. The electrodes were collapsed into 15 clusters according to their caudality and laterality (Table 1). A 2 (anchor: high, low) × 5 (caudality: F, FC, C, CP, P) × 3 (laterality: left, middle, right) three-way repeated measures ANOVA was applied to the cluster mean ERS. The Greenhouse-Geisser correction was applied for the violation of the sphericity assumption in ANOVA (uncorrected degrees of freedom are reported with corrected *p*-values and epsilon values (*ε*)), and multiple comparisons were corrected with the Bonferroni method when appropriate.

**Table 1:** Electrode clusters for theta band ERS repeated-measures ANOVA.

Clusters		Laterality		
		Left	Middle	Right
Caudality	F	AF3, F7, F5, F3	F1, Fz, F2	F4, F6, F8, AF4
	FC	FT7, FC5, FC3	FC1, FCz, FC2	FC4, FC6, FT8
	C	T7, C5, C3	C1, Cz, C2	C4, C6, T8
	CP	TP7, CP5, CP3	CP1, CPz, CP2	CP4, CP6, TP8
	P	P5, P3	P1, Pz, P2	P4, P6

## Results

### Behavioral results

The analysis identified a significant effect of anchor on the subjects’ WTA for the noise (F_(2, 36)_ = 29.881, *p* < 0.001). The post hoc pair-wise comparisons indicated that the average WTA was significantly higher for the high anchor condition (M = 14.178, SD = 1.233) than for the low condition (M = 10.576, SD = 0.987; *p*_high, low_ < 0.001), and the WTA in the medium condition (M = 12.505, SD = 1.102) fell between the high and low conditions with significant discrepancies (*p*_high, medium_ = 0.001, *p*_medium, low_ = 0.002). The effect size as percentage was 29.10% and the effect size as Cohen’s d was 0.744.

### ERP results

**Fig 2:**
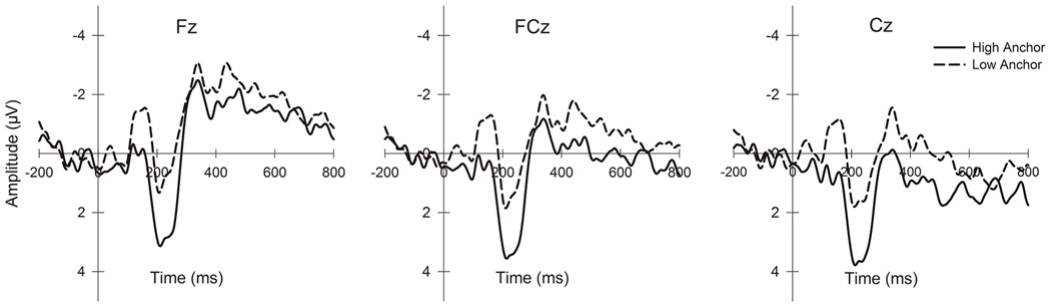
Grand-average P2 waveforms from channels Fz, FCz and Cz in two anchor conditions (high, low) time-locked to anchor onset.

**Fig 3:**
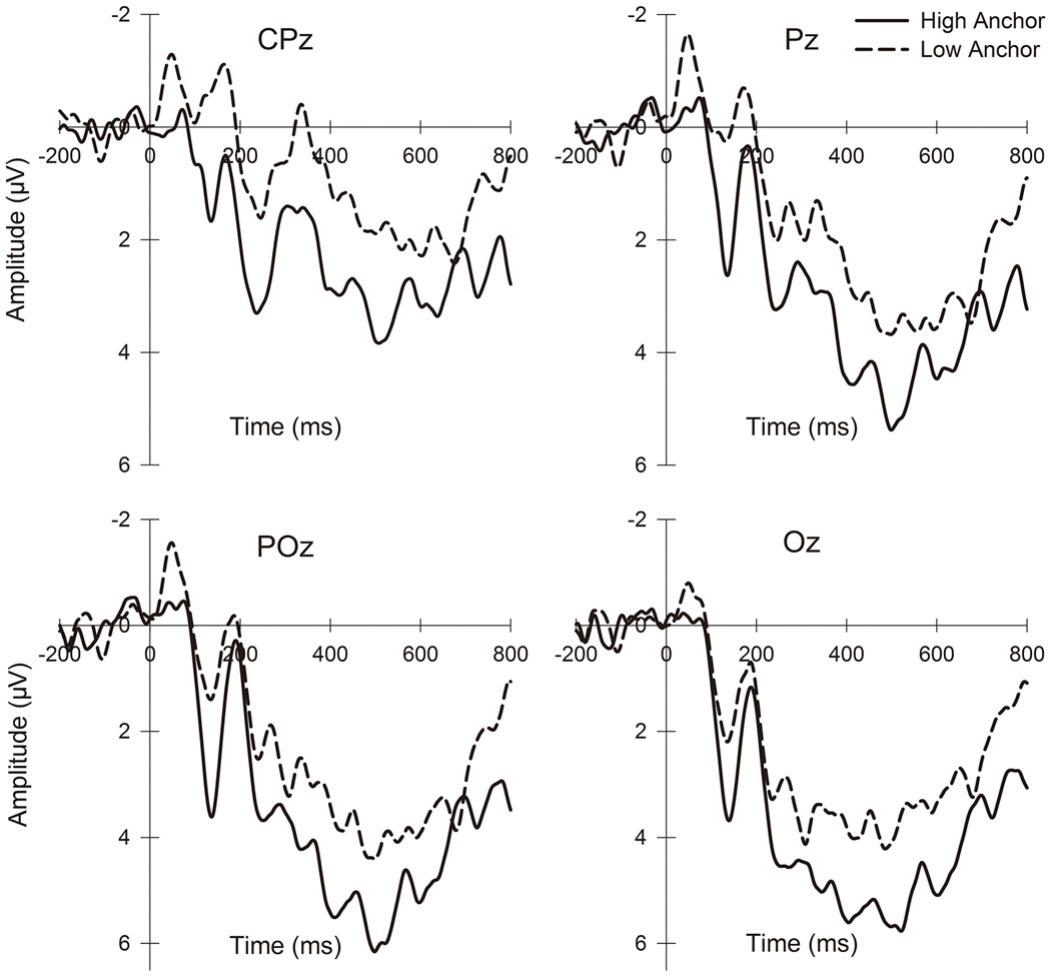
Grand-average LPP waveforms from channels CPz, Pz, POz and Oz in two anchor conditions (high, low) time-locked to anchor onset.

The grand average ERPs under the three anchor conditions are presented in Figs 2 and 3. Repeated-measures ANOVAs of the mean amplitudes of P2 identified a significant main effect of the anchor (F_(1, 18)_ = 15.368, *p* = 0.001), whereas no main effect of the other factors or interactions of the factors was identified (caudality: F_(2, 36)_ = 2.324, *p* = 0.139, *ε* = 0.597; laterality: F_(2, 36)_ = 1.903, *p* = 0.164; anchor × caudality: F_(2, 36)_ = 0.334, *p* = 0.718; anchor × laterality: F_(2, 36)_ = 2.117, *p* = 0.135; caudality × laterality: F_(4, 72)_ = 0.400, *p* = 0.704, *ε* = 0.582; anchor × caudality × laterality: F_(4, 72)_ = 0.825, *p* = 0.456, *ε* = 0.552). The high anchors induced larger P2 amplitudes relative to the low anchors. The statistics for the mean LPP amplitude indicated similar results: only the main effect of anchor (F_(1, 18)_ = 7.289, *p* = 0.015) was significant; the main effect of the other factors and the interactions of the factors was not significant (caudality: F_(3, 54)_ = 1.911, *p* = 0.182, *ε* = 0.382; laterality: F_(2, 36)_ = 0.998, *p* = 0.379; anchor × caudality: F_(3, 54)_ = 0.331, *p* = 0.699, *ε* = 0.602; anchor × laterality: F_(2, 36)_ = 0.244, *p* = 0.785; caudality × laterality: F_(6, 108)_ = 0.400, *p* = 0.366, *ε* = 0.621; anchor × caudality × laterality: F_(6, 108)_ = 1.314, *p* = 0.257). LPP amplitudes were larger in the high anchor condition.

The mean amplitude of the N1-P2 complex elicited by the noise did not differ significantly between the high and low anchor conditions (F_(1, 18)_ = 2.689, *p* = 0.118). However, both factors of the electrode distribution had significant effects on the N1-P2 complex amplitude (caudality: F_(4, 72)_ = 23.079, *p* < 0.001, *ε* = 0.323; laterality: F_(2, 36)_ = 66.607, *p* < 0.001). The amplitude was larger in the central region and the midline and reached a maximum at Cz. No interaction effects were identified (anchor × caudality: F_(4, 72)_ = 1.291, *p* = 0.278, *ε* = 0.310; anchor × laterality: F_(2, 36)_ = 1.226, *p* = 0.294, *ε* = 0.663; caudality × laterality: F_(8, 144)_ = 2.908, *p* = 0.076, *ε* = 0.218; anchor × caudality × laterality: F_(8, 144)_ = 0.510, *p* = 0.615, *ε* = 0.265).

### Band power results

**Fig 4:**
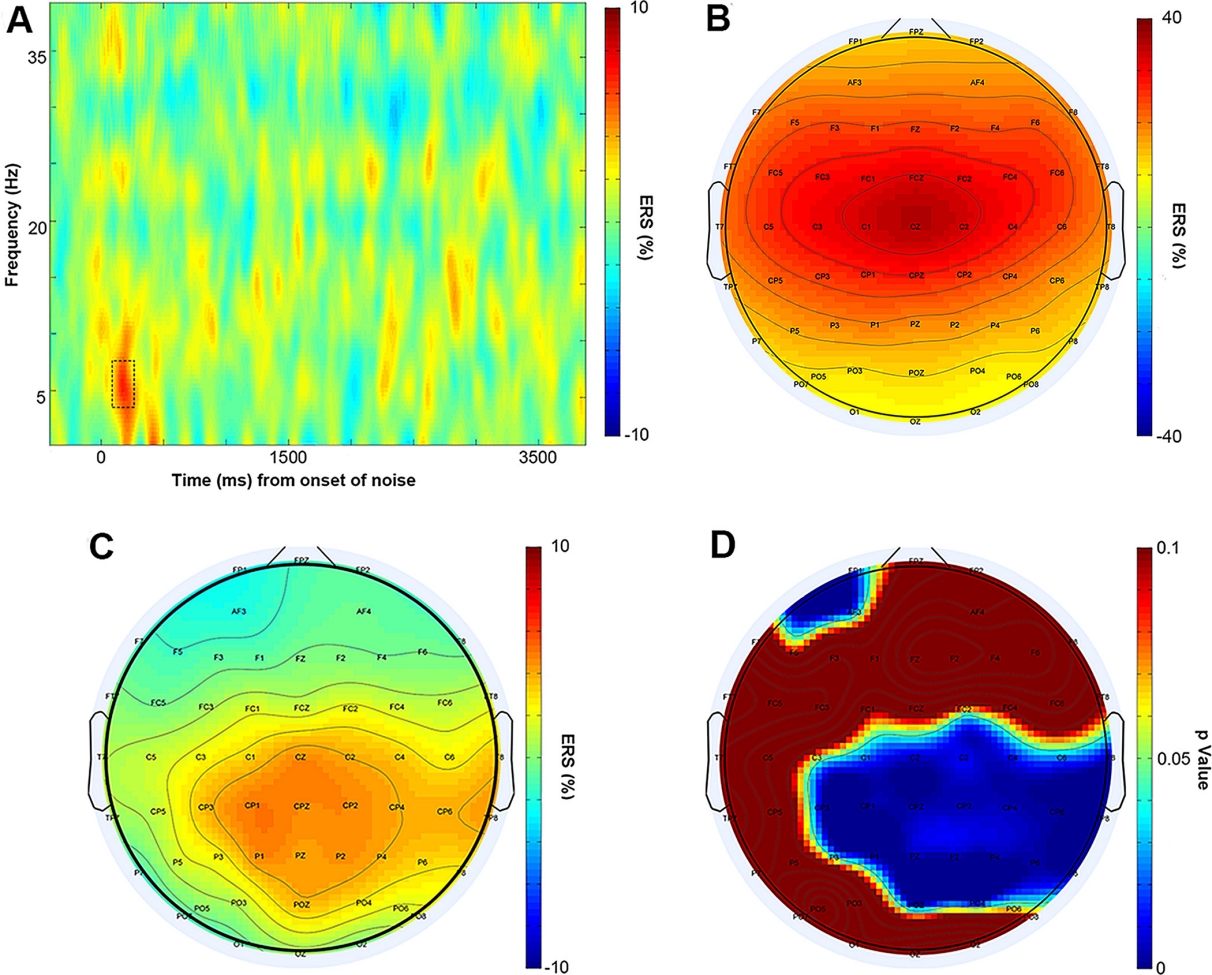
Theta band ERS differences. (A) The time-frequency map shows the subtraction of the ERS of the low anchor condition from that of the high anchor condition over time (x-axis; 0 is onset of the noise stimulus) and frequency (y-axis) at Cz where ERS reached its maximum. The dotted contour corresponds to the window of 80–280 ms, 4–8 Hz from which the topographical map data in (B), (C) and (D) were obtained. (B) Average theta band ERS (high and low anchors) scalp map. (C) Difference in the theta band ERS (high anchor minus low anchor) scalp map. (D) Scalp map of the *p*-value of the one-tail t-test (high anchor > low anchor) for the theta band ERS. Blue colors indicate *p* < 0.05.

The theta band ERS results are illustrated in Fig 4. A significant main effect of the anchor on the theta band ERS shortly after noise onset was observed (F_(1, 18)_ = 4.576, *p* = 0.046). The noise pieces in the high anchor condition induced a larger ERS in the theta band than those in the low anchor condition. Moreover, the effect of scalp distribution on theta oscillatory activities was evident because the main effects of caudality (F_(4, 72)_ = 23.747, *p* < 0.001, *ε* = 0.417) and laterality (F_(2, 36)_ = 18.793, *p* < 0.001) were both significant (Fig 4). The post hoc pair-wise comparisons with Bonferroni correction indicated that the theta powers were significantly larger in the frontocentral (FC, C) than the other (F, CP, P) regions (*p*_FC,F_ < 0.001, *p*_FC,CP_ = 0.007, *p*_FC,P_ < 0.001; *p*_C,F_ = 0.024, *p*_C,CP_ < 0.001, *p*_C,P_ < 0.001). The effect of laterality indicated that the theta ERS in the midline was significantly larger than those in the left and right hemispheres (*p*_middle, left_ = 0.004, *p*_middle, right_ < 0.001); however, there was no significant disparity in the ERS between the left and right hemispheres (*p*_left, right_ = 0.485).

**Fig 5:**
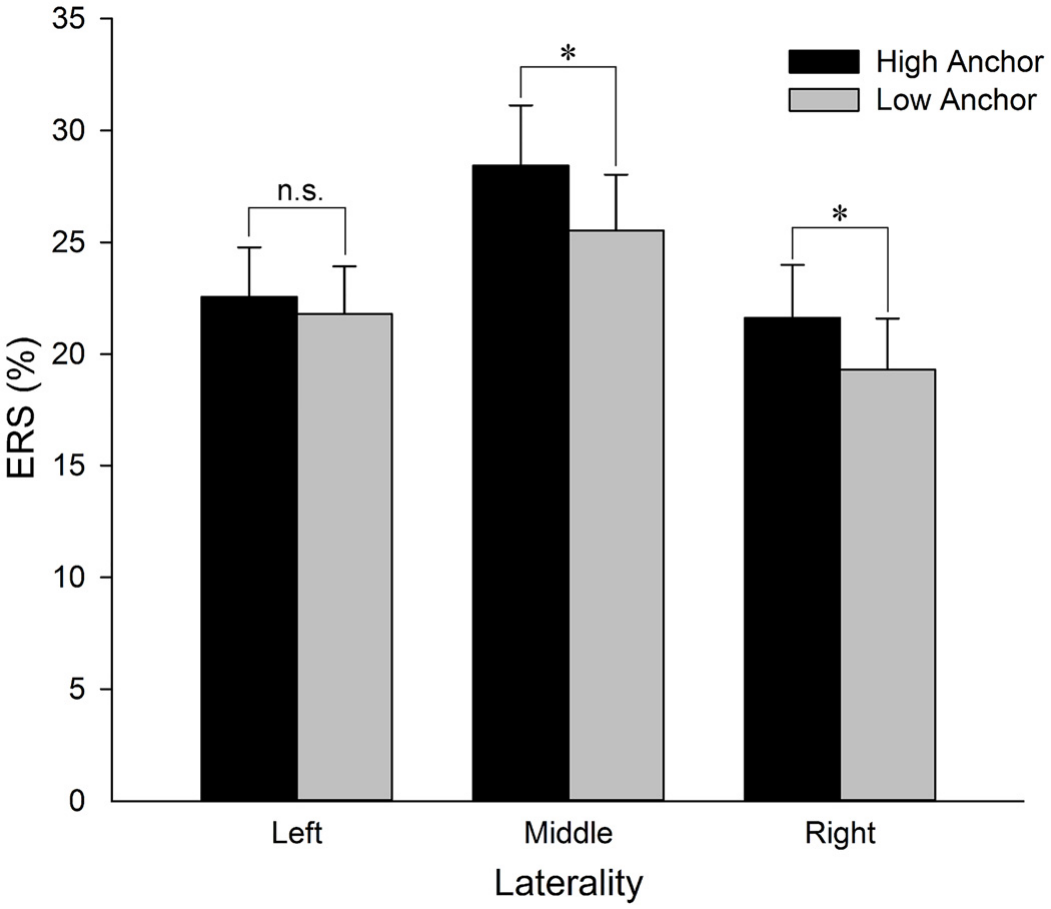
Comparisons of the theta band ERS when the noise was played between the high and low anchor conditions on the left, middle and right hemispheres. Data were collapsed within five caudalities (F, FC, C, CP and P). Asterisks indicate significant (*p < 0.05) differences between the high and low anchor conditions.

The interaction effect between the anchor and caudality was significant (F_(4, 72)_ = 10.320, *p* < 0.001, *ε* = 0.493). An additional simple effect analysis suggested that the theta ERS difference under the two anchor conditions was significant from the central to posterior regions (C: F_(1, 18)_ = 6.44, *p* = 0.021; CP: F_(1, 18)_ = 9.13, *p* = 0.007; P: F_(1, 18)_ = 8.07, *p* = 0.011); however, it was not pronounced in the more anterior regions (F: F_(1, 18)_ = 0.70, *p* = 0.415; FC: F_(1, 18)_ = 1.02, *p* = 0.325). There was also a significant interaction between the anchor and laterality (F_(2, 36)_ = 3.352, *p* = 0.046). A simple effect analysis demonstrated that the ERS was significantly different in the midline and on the right hemisphere (middle: F_(1, 18)_ = 6.18, *p* = 0.023; right: F_(1, 18)_ = 6.64, *p* = 0.019). The interaction between the anchor and laterality is illustrated in Fig 5.

In addition, a significant interaction effect between caudality and laterality was observed (F_(8, 144)_ = 10.661, *p* < 0.001). However, a three-way interaction was not observed (F_(8, 144)_ = 0.778, *p* = 0.537, *ε* = 0.473).

## Discussion

Although substantial research has demonstrated the omnipresence of anchoring effects, whether arbitrary anchors exert effects on consumer WTA for hedonic experiences remains controversial (see debates: [13,14]. Here, we utilized annoying sounds as judgment targets to investigate anchoring effects. Because WTA for listening to annoying sounds was novel to the subjects, there were no established market prices for this experience, and the subjects had a limited ability to use a specific pricing strategy based on previous knowledge or experience [11]. Moreover, the noise comprised a simple sensory experience with few confounding factors, such as utilitarian aspects or aesthetics, which are typically tied to common consumer goods. Finally, there was little satiation or sensitization throughout the repeated trials [2]. Thus, this paradigm allowed us to explore the potential effects that could be predominately contributed to the anchor. Consistent with Ariely et al. [2], our study demonstrated that the subjects’ WTA for noise pieces were assimilated to randomly drawn numbers. First, the statistics indicated that the WTA was significantly larger in the high anchor condition than the low condition. Second, to compare with previous studies, we further analyzed the anchoring effect size using the methods of Simonsohn et al. [14]. The effect size was lower than that observed by Ariely et al. [2] but higher than that reported by Maniadis et al. [13]. This effect size can be considered sizable according to Simonsohn et al. [14]. The effect size based on Cohen’s d also suggests that the anchoring effect in our study approaches a large size (i.e., Cohen’s d = 0.8, [14]. These tests further validate our findings and increase their comparability to existing studies. Thus, our research provides evidence of the robustness of the anchoring effect in the domain of economic value construction. Since initially demonstrated by Brown (1953, as cited in [20], the anchoring effect has been considered to be among the most striking phenomena regarding value formation [61], and our research has again verified its universality in a specific judgment domain. From another perspective, in the case of monetary valuation, prices attached to certain products or experiences can be affected by non-economic but psychological factors, including reference positions in the endowment effect [62], as well as irrelevant information in the anchoring effect.

In the investigation of the potential cognitive mechanism of the anchoring phenomenon, the EEG data suggested that random numbers affected the subjects’ final judgment by acting as primes. The activated semantic information of these anchors biased the WTA because it primed both the subjects’ expectations for the subsequent hedonic experience and the actual perceptions of the experience. First, higher anchors induced larger frontal to central P2 and posterior LPP amplitudes when the subjects viewed the numbers. In our experimental settings, these two components can be interpreted as indicators of the extent to which the subjects perceived the aversiveness of the subsequent experience, consistent with several ERP studies [35–38,56]. Prior to exposure to images with negative emotional valence, increased P2 amplitudes were observed in the subjects with a high intolerance of uncertainty, suggesting that P2 is associated with cue-evoked worry [55]. A positive correlation between P2 amplitude and threat anticipation intensity was also observed in a study of children [63]. In viewing picture cues for potential subsequent electric shocks, the threat-of-shock pictures induced enhanced P2 and LPP amplitudes [38]. Earlier ERP research regarding the emotional and attentional aspects of P2 and late positive components has suggested that more affectively intensive stimuli elicited larger amplitudes because they recruited more attention [64–67]. This finding may explain the association between these two ERP components with various emotion-loaded stimuli in a substantial body of research. Therefore, it can be implied from our results that the semantic information of anchor numbers was activated and led to anchor-consistent anticipatory emotions, i.e., larger numbers indicated that more annoying noise pieces would follow. Moreover, existing studies have generally used explicit cues to inform subjects of the upcoming emotional stimuli; these cues consequently induced P2 or LPP because of their established associations with certain emotions in the subsequent events. However, here, the elicitation of the same ERP components should be primarily attributed to the activated anchors’ semantic information, which shaped the subjects’ anticipation. Based on previous findings regarding the relationship between anticipation and P2/LPP, these ERP components should demonstrate the association of the anchors, perhaps implicitly but strongly, with the aversive upcoming experience, and that the degree of anticipatory aversiveness corresponded with the semantic information activated by these anchors.

Second, the noise pieces induced theta band ERS, which was most pronounced in the central regions and along the midline, and the noise pieces following higher anchors induced an overall larger ERS compared with those following lower anchors. Furthermore, the theta band ERS difference was more pronounced in relatively posterior areas and lateralized to the right. Theta band ERS is thought to reflect human brain activity enhancement by stimuli that are more arousing or attract more attention, as suggested by Başar et al. [40] as “‘Orienting’—a coordinated response indicating alertness, arousal or readiness, is related to theta oscillations…”. Previous studies of affective visual [43–46] or audio [47] stimuli and dislikeness ratings in consumer judgment [48] have suggested that larger theta band power in cortical regions, including the frontal, central and parietal lobes, can be interpreted as an indicator of higher arousal of negative emotion, and our data indicate that anchors also alter subjects’ unpleasantness toward the noises they are subjected to. In addition, the theta ERS discrepancy between the high and low anchor conditions was significant on the right but not the left hemisphere. Previous research has suggested that power enhancement in the right frontal cortices may represent the neural correlates of defensive or withdrawal motivation [68–70]. In alignment with this hemisphere theory, several studies have identified larger overall theta band power of more negative affective responses in the right hemisphere [47,48]. Thus, the larger ERS in high compared with low anchor conditions over the right hemisphere may further indicate more negative experiences when subjects listened to noise following higher numbers in this experiment. This interpretation is supported by a study that demonstrated greater activation in the right hemisphere in response to negative cues in emotion perception [71]. Within the domain of secondary reward value coding, studies have also provided evidence for a correlation between theta band ERS and monetary valuation. Enhanced theta band synchronization has been demonstrated to be related to monetary losses during the feedback stage of gambles [72–74], which indicates that theta power may denote brain coding for negative outcome valuation. Our results may also suggest a similarity of brain coding between negative primary and secondary values. There was no difference between the two anchor conditions in the typical auditory evoked potential N1-P2 complex, which was a vertex potential consistent with previous research [75]. This result indicates that there was no distinction between the spectral and temporal properties of the noise pieces played in the two conditions [59,76], and differences in annoyingness should not be ascribed to physical factors, such as frequency and loudness. The EEG evidence suggests that anchors modulated the neural basis of the formation of the hedonic experience valuation. During the process of target judging, previously activated semantic information consistent with the anchors remained strong and accessible to prime the subjects’ judgments in an assimilatory way.

These EEG results support the semantic priming model of anchoring effects on WTA, in which activation of anchor-consistent semantic information is increased and a judgment is formed overly based on this more accessible type of information [21]. By directly observing the EEG when the subjects encountered arbitrary anchors and were exposed to the judgment targets, we demonstrated that the anchor numbers were immediately processed as primes that activated various levels of anticipatory negative emotion, even though the subjects had not begun to utilize the anchor numbers as a price reference within the time interval of several hundred milliseconds. Semantic information may have anchored the subjects’ WTA as early as in the evaluation stage before the final value elicitation of the target because our data demonstrated that the emotional responses to listening to the noise were consistent with what the anchors had cued. Compared to existing EEG research on anchoring effects [24], we consider it is not probable that the anchors’ influence on WTA can be attributed to an insufficient adjustment process. If the anchor numbers were used as starting points of adjustment, they should not have produced disparity of expectation to the noise as reflected by the ERP components here under different anchor conditions. Neither should they bias the actual feeling, which was indicated by the ERS difference, when subjects underwent the painful experience. Furthermore, as put forward in previous anchoring research [77], the adjustment process itself is likely to be the same in both high and low anchor conditions. Thus, even if the adjustment exists in the valuation formation or presentation on an explicit scale and is represented by the EEG data, there should be no significant differences of the EEG indices under the two anchor conditions. Clearly this is not the case in our results. Our EEG results also suggest that the anchoring effect here should not be attributed to a numeric priming process where judgments are simply primed by the numeric magnitudes of the anchors, because we didn’t observe difference in those ERP components (i.e. N1, N2, P2p and P300) that were proved to be related to numeric magnitude processing (cf. [78–81]. Nevertheless, this interpretation must be treated cautiously. In our study, the numeric magnitude is not dissociable from the anchors because they are essentially numbers. Therefore, future research is still required to address the comparison of the numeric and semantic priming models. Previous studies have typically inferred the cognitive mechanisms from the anchored judgment results. Researchers have exerted certain manipulations that could change participants’ cognitive styles or information processing and thus modulate the anchoring effect and conjectured the causes of anchoring effects from the results (e.g., [17,82]. Few studies have observed the process of judgment formation [20,83] or brain activities that reflect this process [84]. The current study is the first to primarily address the mechanisms of anchoring effects on valuation using neuroscientific methods to explore the anchoring process.

Moreover, our finding that irrelevant anchors act on WTA via the modulation of subjective hedonic experiences supports the theories of context dependency in human valuation by providing direct evidence of brain activities in the value coding process. Plassmann et al. [32] demonstrated that increasing prices of wines could increase the reported ratings of the pleasantness of tasting, as well as the neural activation in the medial orbitofrontal cortex, an area associated with pleasantness for hedonic experiences. Research regarding the subjective affective rating of odors also demonstrated that the reported pleasantness of the same odor varied when labeled with different names, and the modulation of labels was reflected in the rostral anterior cingulate and medial orbitofrontal cortex activities [85]. Wager et al. [28,86] investigated the placebo effects of pain and demonstrated that the actual pain perception was biologically modulated by placebos, as reflected by the activation in pain-related brain regions (thalamus, insula, and anterior cingulate cortex) and laser-evoked potentials. Similarly, in our experiment, randomly drawn numbers influenced the electrophysiological response induced by aversive stimuli, which suggests that the subjective ratings regarding the hedonic experience represented by WTA were sensitive to external references of different forms. However, the contextual factors in this current research were evidently random numbers rather than attached price tags or labels as in previous studies, which were naturally perceived to possess information of products or hedonic experience. Thus, our findings further imply that this sensitivity to the judgment context remains even when information appears as a very subtle hint.

Researchers have noted that in contrast to classical economic theories, individuals lack an inherent, stable preference and a value scale that is immune to the judgment context and presence of prior cues [87,88]. The human brain often constructs value in a relative manner in which relative coding and adaptive scaling may occur [26]. This study supports previous research by demonstrating the arbitrariness [2] of the hedonic experience valuation created by random anchors. More importantly, the neural processing during the valuation was directly examined to investigate the potential cognitive models that underlie anchoring effects, and the electrophysiological responses suggested that anchors primed the subsequent valuation via the activation of semantic information that both updated prior beliefs and altered the experience of the judgment targets.

## Supporting Information

S1 Dataset Data. (XLSX)

S1 Text Experimental instruction. (DOCX)
