# Supplementary material for: Anchors as Semantic Primes in Value Construction: An EEG Study of the Anchoring Effect
Source: PLoS One. 2015 Oct 6;10(10):e0139954. doi: 10.1371/journal.pone.0139954 (PMC4595290; doi:10.1371/journal.pone.0139954)
Supplement: S1 Text — (DOCX) [file pone.0139954.s002.docx]

**Experimental Instruction**

Welcome to this experiment. The experiment includes two phases.

**Phase 1**

You will listen to 90 short-lasting pieces of noise. Each time after you listening to a piece of noise, you will have to decide the minimum amount of money (P) required for listening to this noise in quadruple volume.

The procedure of each trial is as follows. First, please be concentrated when you see a cross fixation. Second, you will see a rotating clover and you can click the mouse to stop it to draw a random price (a). Later, you will hear a piece of noise playing. When the noise end, you will need to consider whether the minimum compensation P is equal to a and click the corresponding button. If P = a, you can directly click the “Confirm” button to provide your answer. If P ≠ a, please move the bar to the right place on the price axis to denote P and then click to confirm.


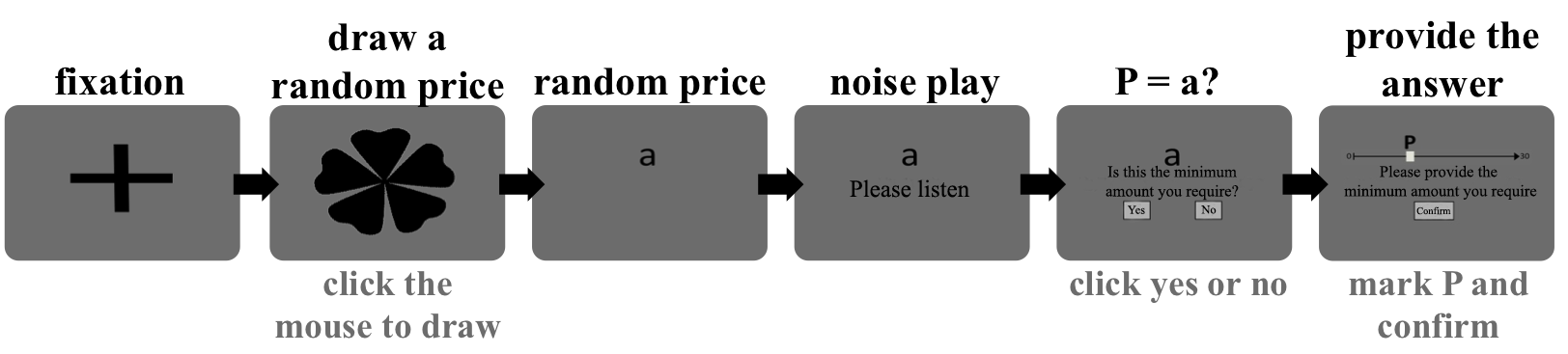


As this pricing task can be quite novel to you, you will need to practice for ten rounds before the main session. In these round, you just need to familiarize yourself with noise pricing and you will not draw any random price or compare. The practice is illustrated as below.


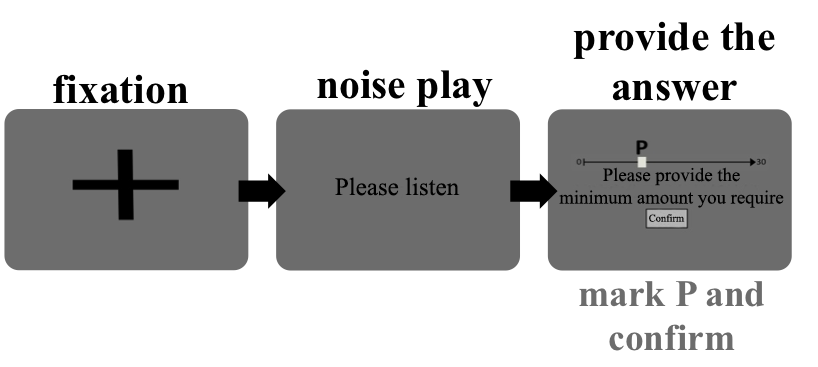


**Phase 2**

After all of the noise pricing, from 90 rounds you have to draw a random round that will count (the practice rounds are not included). On that certain round, whether you will listen to the intensified noise or not depends on the following rules.

1. You will draw a price tag from a paper carton that contains 90 price tags. The prices on the tags range from ￥1 to ￥15. These prices are irrelevant to the random prices you will have drawn by the rotating clover in Phase 1.

2. Let us suppose that the price on the tag you have drawn is X and the minimum amount of compensation you required in the drawn round is P. If X ≥ P, which means that the experimenter can afford the compensation, then you will have to listen to the same noise in that round in quadruple volume and get the compensation of ￥X.

3. If X < P, which means that the experimenter can not afford to compensate, then you will not have to listen to the noise and therefore get no compensation.
